# On Cretinism
*"A Physician's Holiday; or, A Month in Switzerland in the Summer of 1848. By John Forbes, M.D., F.R.S. London: John Murray, and J. Churchill. 1848."


**Published:** 1850-01-01

**Authors:** 


### Art. VI.—ON CRETINISM.*

We make no apology to our readers for extracting from the interesting volume before us the author's account of his visit to Dr. Guggenbühl's establishment for the treatment of cretinism. This is the only portion of Dr. Forbes's Tour in Switzerland that comes within the scope of our Journal. We cannot, however, allow this opportunity to pass without expressing the high gratification we have derived from the perusal of this and other parts of the volume. The work is an admirable hand-book for travellers visiting that portion of the European continent:—

"On arriving at the Institution, I found Dr. Guggenbühl would not be at leisure for a short time; so, leaving my horse at the house, I took the opportunity of mounting the open slope some half a mile higher, in order to inspect the place still more completely. On this short journey I had for companion a young man of Interlachen,

* "A Physician's Holiday; or, A Month in Switzerland in the Summer of 1848. By John Forbes, M.D., F.R.S. London: John Murray, and J. Churchill. 1848."

whom we had overtaken in the forest, and who was now pursuing his further journey over the crown of the Abendberg to the Alpine valleys beyond. He was a saddler, and carried a small pack of leather skull-caps of his own manufacture, for disposal among the cowherds and dairymen. These caps cost about a franc each, and are used by the milkers, who, partly from convenience of posture, and partly with the view of promoting the flow of milk, are in the habit of pressing the head against the cow's flanks while milking. I mention this little circumstance as an evidence of a degree of cleanliness on the part of these people, which they have not always got credit for; and also an illustration of the industrious and simple habits of the Swiss artisans, even at the present day. This young man told me that he might probably be two or three days among the mountains disposing of his goods, during which time he would sleep in the chalets, and live on the produce of the dairies. He seemed well-informed and was very neatly dressed. The day before, we had met at the Hotel of the Jungfrau another young man of the same trade, who had just returned from the United States and the Mexican campaign, with some money in his pocket. He, however, was about to start again for his adopted country, the great republic of the West.

"In descending towards the house, I encountered midway, on the green slopes, some twenty of Dr. Guggenbühl's patients or pupils, climbing the hill for air, exercise, and amusement—all combined—under the superintendence of a well-dressed young man and two of the sisters of charity who belong to the establishment. They were all children, from the age of twelve or thereabouts down to three or four: one was carried by a servant, being incapable of walking. They were running and waddling and tumbling on the grass, and playing in their own way, with the servants, with one another, and with a fine good-natured dog who made one of the party, and who was probably of nearly the some intellectual caliber as some of his poor biped companions.

"This little exhibition at once satisfied me of the enlightened character of Dr. Guggenbühl's views; and I felt much greater pleasure in thus observing and examining the poor objects of his benevolent care, amid their humble enjoyments, and as it were in Nature's own presence, than if I had seen them cooped up in a ward or schoolroom, under restrictions which they probably could neither understand nor well brook. They were all neatly and cleanly but plainly dressed, and, like most individuals of the pitiable class to which they belong, were cheerful and apparently happy. The motherly care shown to them by the excellent sisters was delightful to witness. Sitting down in the sun on the beautiful soft grass, or trooping about you with that social instinct which seems so strong in idiots, with endless shaking of hands and the same monotonous greetings, repeated again and again, they seemed to renew the interesting scene I had so lately witnessed at the congenial establishment on Highgate Hill. And, indeed, I was much more struck with the similarity of the subjects, in the two cases, than I had expected. Several of Dr. Guggenbühl's patients unquestionably presented such characteristics of cretinism, in their dwarfish shape, peculiar configuration of head, and odd, old expression of countenance, as left no doubt as to their class; but there were some of them regarded by Dr. Guggenbühl as cretins, whom my less-practised eye could in no way distinguish from the ordinary idiots of other countries; and there were others whom he himself admitted to be simple idiots, though natives of Switzerland. Two or three of the children had come from districts where cretinism does not prevail: one was an infant from England.

"On my return to the house, I found Dr. Guggenbühl ready to receive me, and to receive me with that cordiality and kindness which form so marked a feature in his character. He showed me over the establishment, detailed his views, submitted to my inspection the most interesting cases, and put in operation before me some of his practical methods for developing both the physical and moral powers of the children.

"Dr. Guggenbühl justly considers cretinism as a physical malady, consisting in an imperfect development of most of the bodily organs, and of the brain in particular, on the imperfection of which latter organ all the mental incapacity depends. Whatever be the special cause of the affection, he concludes that it is only by improving the bodily health generally, by strengthening and improving—that is, developing to a higher degree of functional activity—all the organs of the system, and among the rest, and in an especial manner, the brain, that any rational hope of benefit can be founded. It was therefore a beautiful and most philosophical principle which he adopted as the indispensable basis of all his practice—that, namely, of having the infant-cretin removed from the low, close valleys in which the malady generally originates, to the free, dry, cool, bracing air of the open yet comparatively sheltered and sunny slopes of the Abendberg. In such a locality, which in itself is powerfully restorative, all the most effectual means of improving health can be applied with the best prospect of success; while they must have as obviously failed so long as the deteriorating causes were in full operation around.

"These restorative means are the few simple ones which medical science has long recognised as alone influential—plenty of good food, exercise in the open air, friction to the surface, bathing, moderate mental exercise, cheerful occupation, comfort and happiness, with such small auxiliaries as medicine can supply in the form of drugs to promote healthy and correct unhealthy actions. The attempt to teach or to improve the mind in such cases, without improving the whole animal system at the same time, would be absurd, and could only be thought of by men who are utterly ignorant of human nature, that is, of animal nature. It would be as rational to try to extract music from a violin without strings, or with slack strings, and with a cracked and broked frame, as to seek to develope and improve the mind of a cretin without previously strengthening and mending, and otherwise improving the qualities of his mental organ—the brain.

When this is done, or even when this is doing, attempts may safely and with propriety be made to rouse the mental faculties and to improve and develope them; in other words, to teach: but not before. And it is by pursuing this natural, physiological system, heedless of the visionary dogmas of the metaphysicians, that Dr. Guggenbühl has already attained most gratifying and important results in the treatment of his patients and pupils.

Dr. Guggenbühl justly regards *all* the external influences with which his pupils are surrounded as of more or less importance in developing their slumbering faculties, well knowing that the restorative or curative power, which is but another name for nature, can only work efficiently when it works leisurely, uninterruptedly, and for a long period. It is, therefore, not without good reason that in selecting the Abendberg for his residence, he took into consideration, not simply its air and sun, its dryness, its sheltered exposure, and its facilities for exercise, but also its local charms, the beauty and grandeur of the scenes which lie around it, and force themselves incessantly on the senses of the pupils, without any effort on their part or that of others. The green valley of Interlachen with its lofty yet living barriers of mountain forest, the series of mountains that stretch from this in every direction, the lakes of Brientz and Thun with their connecting stream, and, above all, the grand chain of the Oberland, with its snowy peaks piercing the blue sky close at hand—all these cannot be supposed to be without effect in exciting attention and interest, that is, in stimulating the material of the mind to action, and action of the best kind.

Heroic doctors, in their ignorance of the way in which alone Nature works, may attempt to cure a chronic disease by a *coup de main*; and, by mistaking temporary relief for real cure, may themselves suppose, or be supposed by others, to have done so; but every physiological physician knows well, that a morbid condition which may have been months or years in forming, can only be effectually and permanently removed by means which act slowly and for a length of time, not on one part only, but, more or less, on the whole system. And so it is, and still more certainly, in the cases now under consideration; in the cure or amelioration of which nothing is to be neglected that can help to waken up the dormant faculties, in that gentle and imperceptible but uninterrupted mode in which Nature produces all her great and permanent changes in the organic world of life, and in the psychical no less than in the mere physical portion of her domains.

But Dr. Guggenbühl is far from trusting to these general influences alone, or to means directed to the improvement of the physical powers of the system at large; in conjunction with these he is in the constant employment of measures intended to act directly in developing the mental faculties. These comprehend everything which is usually included under the term education. When of a fitting age, all his pupils must attend the schoolroom for certain short periods of the day; and there they are carefully disciplined by his teachers and by himself, in exercising their feeble faculties of thought, and in acquiring such small modicums of knowledge as their respective capacities can grasp. The alphabet of letters and figures, syllabification, numeration, writing, outline or diagram drawing, spelling, reading, and such-like elementary processes, are among their first attempts; while to those more advanced, a knowledge of things is communicated in that simple and natural method, by direct action on the senses, or demonstration, which it is to be hoped will ere long entirely supersede, in schools for the rational also, that absurd system of teaching by the ear or by rote, which is no teaching at all, or a teaching only of sounds not ideas.

"Dr. Guggenbühl was so kind as to examine, in my presence, three or four of his more advanced pupils in this species of instruction; and it was delightful at once to see the amount of real knowledge that had been thus acquired, and the gratification which its acquisition and the conscious possession of it evidently conferred on the respective pupils. Not that the poor children knew much or could do much; far from it; but what they did, sufficed to show that the instrument was capable of action, and left no grounds for doubting that perseverance in the same course would lead to something still better. At the very least, the actual result showed the existence in the poor children of the quality of *teachableness;* and this quality can be made subservient, in many ways, to the acquisition of *habits* which cannot fail to add to the comfort, health, and happiness of themselves as well as of their relations. This has been sufficiently manifested by many cases at present in the establishment, and by others that have left it. And, indeed, when we merely see that those who could neither walk, nor talk, nor feed themselves, have learned to do so by instruction, we need not doubt that improvement may take place in matters involving only a little more cerebral or mental action.

"The institution on the Abendberg is the private property of Dr. Guggenbühl, and originated in his own benevolent desire to benefit this wretched class of his countrymen. He has received some assistance, but not much, from the government of Switzerland and from individuals; but his exertions are obviously cramped by inadequate means. He has only about thirty patients at present; but the house is capable of accommodating many more. As Dr. Guggenbühl's method is just as applicable to the improvement of the common idiot—although in simple idiotism accompanied with a good corporeal development, the obtainable results will be relatively less—he does not refuse to admit such into his establishment; and I am sure I am doing a great service to parents afflicted with any offspring of this kind, in recommending the Abendberg to their especial notice.

"As I have already stated, I differ somewhat from my excellent friend the superintendent, in regard to the special nature of some of the cases now in this house; believing that more than one of those he regards as cretins, are in no respect different from the ordinary idiot of this and other countries. And in the course of my examination of such reputed subjects in different parts of Switzerland, I found some among them who, I think, should be classed in the latter category and not the former. This is, indeed, what might be expected. One hears little or nothing of simple idiotism, as distinguished from cretinism, in those districts where the latter malady is known to prevail : yet surely we may reasonably expect the ordinary proportion of such cases ; there being no country in which idiotism does not prevail to a certain extent. I infer that all Swiss idiots are classed as cretins.

"This is not the place to enter upon the consideration of the causes of this great national affliction. They are, in fact, very imperfectly known. The most that can be said on this head is, that the more general features of the localities in which the disease is chiefly found, and the more general external circumstances amid which it occurs, may be considered as ascertained with some approach to accuracy. But even here we want positive proof; and we have no proof whatever as to the actual efficient or immediate cause of the affection. Every theory hitherto advanced is defective as not applying to all the cases.

"It is by no means proved that cretinism has any essential connexion with goitre, beyond the general fact that they both commonly prevail in the same localities in Switzerland. Innumerable goitrous subjects, however, even here, have no taint of cretinism, either as to the general imperfect physical development, or the special cerebral defect; and many cretins have no goitre. This disease, moreover, under the denomination of Bronchocele, is well known to prevail in most countries of the world, chiefly in the valleys of hilly districts, where yet no cretinism is found. It is very common in England. The well-known localities of cretinism as well as of goitre, in Switzerland, are the deeper valleys and their outlets, and it has been very generally admitted, of late, on the strength of little more than this fact of locality, that the main cause there operative in producing them, is what is vaguely termed *confined air*, assisted by the dirty habits of the people, &c. The old theories deriving these diseases from snow-water, water having mineral impregnation, &c., have been generally abandoned as untenable. The water-theory is of all others most improbable, seeing that snow, or glacier-water is most rarely, if ever, used, while that which is in habitual use is among the finest and purest in the world, and exists in boundless quantity.

"Notwithstanding what has just been stated, of the want of evidence as to the essential connexion of goitre and cretinism, I am myself inclined to believe that they may very possibly have an essential connexion, inasmuch as they may be both owing to the same cause, or to a modification of the same cause. I make no attempt to investigate the precise nature of such cause, nor do I propound even a conjecture respecting it, as the result of any special or enlarged consideration of the subject; but my present impression is that it is some form of that unknown local influence or thing, commonly recognised under the name of *miasma* or *malaria*, and which operates on the animal system as a poison, producing special modifications of function and special changes of structure, according to certain special conditions which, however, are like itself unknown. As the unknown thing which we term malaria or miasm of marshes, under certain circumstances, gives rise at one time to simple ague, at another to a fatal remittent fever, &c., and produces at times a morbid enlargement of the spleen, at others disease of the liver, &c. &c.; so I can imagine that some other *malaria* or unknown thing or influence, of local origin, may be the cause of ordinary bronchocele, of the aggravated bronchocele or goitre of the Alps, and also of cretinism. There must be something more than stagnant or impure or heated air requisite for its production, as we find it frequently more rife in the larger than in the smaller valleys, as in the instances of the great valley of the Rhone and the valley of Aosta, which may be regarded almost as its head-quarters. Mere elevation above the sea, however, does not seem to exempt from it, provided the relations of mountain and valley still characterize the locality. Professor Forbes found the disease extremely prevalent on the south side of the Pennine Alps, in the Valpelline, at the elevation of 4000 English feet above the sea.*

"It cannot, therefore, be questioned, under every view of the case, and even while we are still in total ignorance as to its efficient cause, that, in attempting the relief or cure of cretinism, removal from the source of its origin is a most important if not an altogether essential proceeding. I had myself sufficient evidence, not merely of the special preference of this affection for certain localities, but of the actual power of certain localities to produce it in families not otherwise predisposed to it. At Bonneville in Savoy, one of the surgeons pointed out to me a village near that town in a gorge of the mountain-range that bounds the Arve on the south, as the only place where cretinism prevails in that district; and he informed me that he knew a family who had had several healthy children while residing in a more elevated spot, and who, on coming to reside in this village, gave birth to several cretins: and similar instances have been mentioned to me by others, and are also noticed by writers on the subject. I saw cretins in the valley of the Arve, in the valley of Aosta, in the Vallais, in the valley of the Visp, in the valley of the Linth, &c.; but wherever I made inquiries respecting the prevalence of cretinism, I uniformly received the same assurance that it was everywhere on the decrease, and in so marked a degree that the belief of its final extinction at no distant date seemed very general. Dr. Grillet, of Sion, the very intelligent physician to the cretin hospital in that city, and who has made extensive statistical inquiries respecting this disease, regards the gradual diminution of cretinism as fully established.

"The most marked evidence I myself obtained on this point was afforded me by the pastor of St. Nicholas, in the valley of the Visp. The population of his parish is 560 or 570. Among this number

* "Travels in the Alps," p. 271.

there are twelve cretins, all, save one, above the age of thirty; most of them upwards of forty; the exceptional case is seven years of age, and is itself the child of a cretin. It follows from this that there have been very few cretins born in the parish during the last thirty years. The pastor assures me that the child just mentioned is the only one born during the twenty years of his incumbency. He attributes the recent comparative immunity to the change of habits and manners that have taken place of late years. He says, the persons of the people, as well as their houses, are much cleaner than they used to be; the rooms better aired; and that the children are sent much more out of the valley into the mountains, there being now many more alpine chalets for summer residence than was the case formerly.

"In my numerous inquiries respecting cretinism in Switzerland and Piedmont, I was much struck with the illustration it afforded of the influence of habit in modifying some of our strongest instincts and feelings. The mother of an idiot, even in the lowest class, in England, feels the imperfection of her child to be the greatest of personal misfortunes; she looks upon the affliction as of too awful a nature to be thought of except with a solemn humility; and does all in her power to conceal it from the world. I saw nothing of this in Switzerland. On the contrary, the neighbours and companions, and relatives, sometimes even the fathers and mothers of cretins, had not only no delicacy in showing them, but seemed to make their unhappy oddities a subject of mirth: they appeared to regard my inquiries and personal examination of the poor creatures as something essentially odd and ludicrous. I was really shocked to see and hear what I did on these occasions; but on reflection I was not much surprised: custom has in all times been found capable of smothering not only the natural instincts and feelings, but reason itself, even in the minds of the strongest."

---

